# Post-Bariatric Surgery Abdominoplasty Ameliorates Psychological Well-Being in Formerly Obese Patients: A Cross-Sectional Recall Study

**DOI:** 10.3390/jcm14124025

**Published:** 2025-06-06

**Authors:** Krzysztof Drygalski, Ewa Płonowska, Zuzanna Razak Hady, Paulina Głuszyńska, Hady Razak Hady

**Affiliations:** 1 Department of Hypertension and Diabetology, Medical University of Gdansk, Mariana Smoluchowskiego 17, 80-214 Gdańsk, Poland; 2 Department of Anaesthesiology and Intensive Care, Medical University of Bialystok, M. Skłodowskiej-Curie 24A, 15-276 Białystok, Poland; 3 Department of Human Anatomy, Medical University of Bialystok, Mickiewicza 2A, 15-230 Białystok, Poland; 4 1st Clinical Department of General and Endocrine Surgery, Medical University of Białystok, M. Skłodowskiej-Curie 24A, 15-276 Białystok, Polandhadyrazakh@wp.pl (H.R.H.)

**Keywords:** abdominoplasty, sleeve gastrectomy, major weight loss, obesity, quality of life, depression, anxiety

## Abstract

**Background:** Bariatric surgery is an effective treatment for obesity, leading to significant weight loss and improvements in metabolic health. However, massive weight loss often results in excess skin, which can negatively impact body image, psychological well-being, and quality of life. Abdominoplasty is commonly performed after bariatric surgery to address these concerns. Our study aimed to evaluate the effects of post-bariatric abdominoplasty on psychological well-being, body image, social relationships, and sexual functioning in formerly obese patients. **Methods:** A single-center, cross-sectional recall study was conducted on 35 patients, out of 135 invited, who underwent sleeve gastrectomy followed by abdominoplasty 12–24 months after the initial surgery. Participants completed validated questionnaires assessing psychological well-being, depression, anxiety, self-esteem, body perception, social relationships, and sexual functioning. Pairwise comparisons were performed to assess changes across the preoperative, post-bariatric, and post-abdominoplasty stages. **Results:** Psychological well-being significantly improved post-bariatric surgery, with further reductions in anxiety and depressive symptoms after abdominoplasty. However, body shape perception and self-esteem improved after bariatric surgery but did not show additional enhancement following abdominoplasty. Social support remained largely unchanged, except for modest improvements in attachment and reliable alliance. Sexual functioning improved significantly after bariatric surgery but showed no further significant gains after abdominoplasty. **Conclusions:** While abdominoplasty is associated with additional psychological benefits, particularly in reducing anxiety and depressive symptoms, it does not significantly enhance body perception, self-esteem, or sexual functioning beyond the effects of bariatric surgery. These findings highlight the importance of setting realistic patient expectations regarding the benefits of body contouring surgery in post-bariatric care.

## 1. Introduction

Obesity is a widespread and growing concern in many developed countries. High-calorie diets, sedentary lifestyles, and environmental factors all contribute to excessive weight gain, which can lead to serious health complications such as cardiovascular disease, diabetes, and joint disorders [1,2,3]. However, advancements in care for individuals with obesity have introduced new challenges in plastic surgery, as patients experiencing significant weight loss over a relatively short timeframe may subsequently require additional surgical interventions.

Although achieving substantial weight reduction after bariatric surgery is welcomed and beneficial, as it improves glycemic control, lowers blood pressure, and reduces mortality, it can negatively affect body aesthetics [4,5]. Losing weight rapidly often leads to significant skin deformities and loss of subcutaneous volume and tissue rigidity, which can have profound physical, psychological, and social consequences. Excessive loose skin may diminish quality of life, as it creates everyday challenges such as skin maceration, difficulty with clothing, walking, and exercise, and can negatively affect body image perception and intimate relationships [6,7].

Advancements in body-contouring surgery (BCS) have substantially broadened the support available to patients who have experienced significant weight loss. At present, abdominoplasty and lower body lift remain the principal techniques for correcting trunk deformities following major weight loss [8]. These aesthetic procedures offer both functional benefits and aesthetic outcomes. However, their overall impact on quality of life and especially sexual well-being in patients with a history of bariatric surgery remains less explored. Thus, the aim of our study was to assess the impact of sleeve gastrectomy and subsequent abdominoplasty on body image perception, mental and sexual well-being in formerly obese patients.

## 2. Materials and Methods

The study was conducted as a single-center retrospective study. Patients who underwent sleeve gastrectomy due to obesity, followed by abdominoplasty at the 1st Clinical Department of General and Endocrine Surgery, Medical University of Białystok, Poland, between 2018 and 2022, were contacted by phone and invited to participate. One hundred thirty-five patients who expressed interest in participating received an informed consent form by mail and were asked to complete a set of questionnaires. However, we were unable to conduct a prospective study. Therefore, the patients were asked to reflect on how they felt before bariatric surgery, after bariatric surgery but before abdominoplasty, and after abdominoplasty, and to answer each questionnaire as accurately as possible based on their recollections of each period.

Inclusion criteria were having undergone sleeve gastrectomy followed by abdominoplasty 12 to 24 months after bariatric surgery. Exclusion criteria included age below 18 years and inability to provide informed consent. Out of the 135 patients who initially agreed to participate, only 35 returned a fully completed set of questionnaires and were included in the analysis. In addition to the questionnaires, clinical data collected from the department’s database included age, gender, maximum weight and BMI, preoperative weight and BMI, medical history, and history of dietary and lifestyle changes.

The study adhered to the principles of the Declaration of Helsinki and Polish law and was approved by the Bioethical Committee of the Medical University of Białystok, Poland (APK.002.435.2021, issued on 18 November 2021). All participants signed written informed consent and had the opportunity to ask questions regarding the study. All questionnaires were administered in Polish.

### 2.1. Surgical Technique

All patients underwent preoperative marking of surgical incisions based on the amount of excess skin and the desired aesthetic result. Incisions were made from hip to hip along the natural suprapubic crease. A flap was then created between the fascia and fat, extending upward to the xiphoid process of the sternum. In the next step, the umbilicus was circumferentially dissected from the flap and left attached to the abdominal wall via the umbilical stalk. The fascia of the rectus abdominis muscle was plicated using long-lasting absorbable sutures. After carefully measuring the raised flap, the excess skin was marked and excised. The superior part of the flap was then reapproximated to the suprapubic incision using multiple layers of sutures. The umbilicus was transplanted to its new position on the remaining flap. Two drains were inserted through small incisions just below the main suprapubic incision to prevent fluid accumulation. Finally, the surgical site was covered with a sterile dressing, and an abdominal binder was applied.

### 2.2. DSM-5 Questionnaires

General psychological well-being was assessed using the Polish adaptation of the DSM-5-TR Level 1 Cross-Cutting Symptom Measure (DSM-5) [9]. This self- or informant-completed questionnaire evaluates mental health domains relevant to a range of psychiatric conditions and was developed by the American Psychiatric Association [10,11,12]. It consists of 23 questions that assess 13 psychiatric domains: depression, anger, mania, anxiety, somatic symptoms, suicidal ideation, psychosis, sleep problems, memory, repetitive thoughts and behaviors, dissociation, personality functioning, and substance use. Each question is rated on a 5-point scale (0 = none or not at all; 1 = slight or rare, less than a day or two; 2 = mild or several days; 3 = moderate or more than half the days; and 4 = severe or nearly every day). Both the total score and scores for each domain separately were treated as outcome variables.

In addition to the DSM-5 Level 1 test, we used the Polish adaptation of the DSM-5-TR Level 2—Depression—Adult and the DSM-5-TR Level 2—Anxiety—Adult (PROMIS Emotional Distress—Anxiety Short Form) questionnaires to provide a more in-depth evaluation of depression and anxiety, which are commonly associated with obesity [9]. Both scales were developed by the PROMIS Health Organization to assess the specific domains of depression and anxiety in adult populations [13]. In DSM-5-TR Level 2—Depression—Adult scale, scores 17–22 were classified as mild depression, 23–32 as moderate depression, while 33 and over as severe depression. In LEVEL 2—Anxiety—Adult scale, scores 16–20 were classified as mild anxiety, 21–27 as moderate anxiety, while 28 and over as severe anxiety.

### 2.3. Patient Health Questionnaire-9

The Patient Health Questionnaire-9 (PHQ-9) was developed by Spitzer and colleagues, with an educational grant from Pfizer Inc. (New York, NY, USA). [14]. It is widely used for diagnosing depression and assessing mental disorders based on DSM-IV criteria. PHQ-9 consists of nine questions, which are essentially the DSM-IV criteria for depression. It allows both the diagnosis of depression and the evaluation of symptom severity [15]. The questionnaire is brief and easy to score, and it has been translated and validated in multiple languages, including Polish [16].

### 2.4. Rosenberg Self-Esteem Scale

The Rosenberg Self-Esteem Scale (RSES) is a widely used instrument for measuring self-esteem in social science research and is available in Polish [17,18]. It employs a 0–30 scoring range, with scores below 15 possibly indicating low self-esteem. Although initially developed for adolescents, it has been extensively validated for use in adult populations. The RSES contains ten items—five positively worded and five negatively worded—which together provide a comprehensive assessment of an individual’s self-worth.

### 2.5. Body Shape Questionnaire

The Body Shape Questionnaire (BSQ), developed by Cooper et al., is a self-report tool designed to assess concerns related to body shape, particularly the feeling of “being fat”. It is available in Polish [19,20]. While it is commonly used in individuals with bulimia nervosa and anorexia nervosa, it is also relevant for people with obesity. The BSQ includes 34 items, each rated from 0 (least concern) to 6 (most concern), yielding a total score between 0 and 204. Higher scores indicate greater concern. Although originally validated in women, the questionnaire can be adapted for men by modifying certain wording, as recommended by the authors.

### 2.6. Social Provisions Scale

The Social Provisions Scale, developed by Cutrona and Russell, evaluates six relational dimensions proposed by Weiss. Participants rate the extent to which their social relationships provide guidance, reliable alliance, reassurance of worth, social integration, attachment, and opportunities for nurturance. Each dimension is assessed using four items: two indicating the presence of the provision and two its absence. Both total and subdomain scores were used as outcome variables.

### 2.7. Changes in Sexual Functioning Questionnaire

The Changes in Sexual Functioning Questionnaire (CSFQ) was developed by Clayton et al. to assess sexual dysfunction and intimate life-related side effects of psychotropic medications [21,22]. The scale is also available in Polish [23]. It serves as a clinical interview tool for diagnosing and managing sexual dysfunction in individuals receiving outpatient psychiatric care. The CSFQ consists of 14 items and offers separate versions for females and males. It is employed in clinical settings and includes subsections that identify each person’s sexual pattern, allowing an assessment of how much sexual change occurs over time. The questionnaire evaluates five domains of sexual functioning: sexual desire, sexual frequency, sexual satisfaction, sexual arousal, and sexual completion. A total CSFQ score for both women and men is calculated by summing Items 1–14. Scores of 41 or below for women and 47 or below for men suggest sexual dysfunction. In addition, five subscale scores, each with established cutoffs, are calculated: pleasure/overall satisfaction (Item 1 ≤ 4), desire assessed by frequency (Items 2 + 3 ≤ 6 for women, ≤8 for men), desire assessed by interest (Items 4 + 5 + 6 ≤ 9 for women, ≤11 for men), arousal/excitement (Items 7 + 8 + 9 ≤ 12 for women, ≤13 for men), and orgasm/completion (Items 11 + 12 + 13 ≤ 11 for women, ≤13 for men). Although Items 10 and 14 count toward the total score, they are not part of any specific subscale. Both the total score and scores for each domain separately were treated as outcome variables.

### 2.8. Global Aesthetic Improvement Scale

The Global Aesthetic Improvement Scale (GAIS) is a straightforward self-reporting scale assessing the aesthetic outcome of a procedure. Patient scores 1–5 based on rating and a brief description that in his/her opinion fits best (1—very much improved, 2—much improved, 3—improved, 4—no change, 5—worse).

### 2.9. Statistical Analysis

All statistical analyses were conducted using GraphPad Prism 8 (GraphPad Software, version 8.4.0 La Jolla, CA, USA). All patients who returned their completed questionnaires were included in the study. Individual responses were scored according to the instructions of each questionnaire and presented as total scores and subdomain scores when applicable. Normality was assessed using the Shapiro–Wilk test. For comparisons, three paired analyses were conducted: preoperative vs. post-bariatric, preoperative vs. post-abdominoplasty, and post-bariatric vs. post-abdominoplasty. Paired *t*-tests were used for normally distributed data, while the paired Wilcoxon test was used for non-normally distributed data.

## 3. Results

Out of 135 patients who declared an interest in participation in the study, only 35 returned completed questionnaires. All included patients had undergone sleeve gastrectomy due to morbid obesity, followed by abdominoplasty 12 to 24 months after the initial surgery. The majority of patients were female (74.3%). The baseline characteristics of patients who did and did not return the questionnaires are summarized in Table 1.

### 3.1. Psychological Well-Being

General psychological well-being significantly improved following sleeve gastrectomy. The total DSM-5 score significantly decreased from the preoperative stage (16 [11–25.75]) to post-bariatric surgery (12 [5,6,7,8,9,10,11,12,13,14]; *p* < 0.001) and further declined post-abdominoplasty (8 [3,4,5,6,7,8,9,10,11,12]; *p* < 0.001). Notably, a significant difference was observed between the post-bariatric and post-abdominoplasty stages (*p* < 0.01). A similar trend was seen in the PHQ-9, where the difference nearly reached statistical significance (*p* = 0.052), suggesting continued psychological improvement following body contouring surgery.

Within DSM-5 subdomains, the majority of them, like depression, anxiety, anger, memory, repetitive thoughts and behaviors, or personality functioning scores, improved significantly after bariatric surgery, with no further significant reduction following abdominoplasty. However, somatic symptoms and sleep problems showed further improvement after abdominoplasty compared to the post-bariatric stage.

Substance use did not change significantly post-bariatric surgery (*p* = 0.072) but decreased significantly post-abdominoplasty (*p* < 0.01). Similarly, mania scores remained stable post-bariatric surgery (*p* = 0.294) but showed a significant reduction post-abdominoplasty (*p* < 0.01) (Table 2).

The results suggest that despite improvements in psychological health being evident at both surgical stages, the most notable changes occurred post-bariatric surgery, with abdominoplasty providing additional benefits in some areas.

### 3.2. Depression and Anxiety

To examine depression and anxiety in greater depth, we used the DSM-5 Level 2 instruments. Depression symptoms, assessed with the DSM-5-TR Level 2—Depression scale, showed significant improvement after both surgeries. Median scores decreased from 10.5 (IQR: 5–17.5) preoperatively to 4 (IQR: 2–7) post-bariatric surgery (*p* < 0.001) and further declined to 3 (IQR: 1–6) post-abdominoplasty (*p* < 0.001). However, the difference between the post-bariatric and post-abdominoplasty scores did not reach significance (*p* = 0.052) (Table 3).

Anxiety severity, measured by the DSM-5 PROMIS Level 2—Anxiety scale, also declined significantly after both procedures. Median anxiety scores dropped from 4 (IQR: 0–12.25) preoperatively to 1 (IQR: 0–5) post-bariatric surgery (*p* < 0.01) and further to 1 (IQR: 0–3) post-abdominoplasty (*p* < 0.001). This additional improvement was statistically significant (*p* < 0.05) (Table 3).

**Figure 1:**
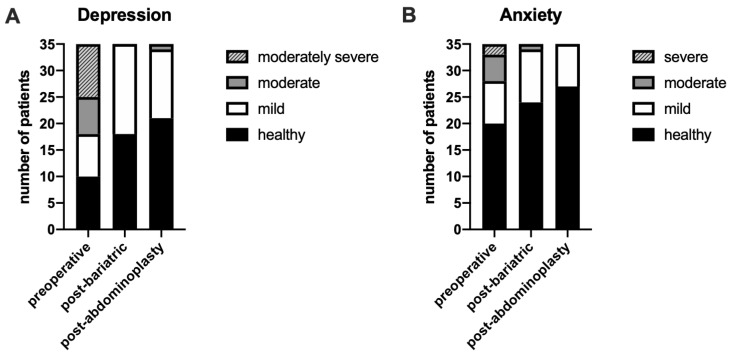
The effect of bariatric surgery and subsequent abdominoplasty on the occurrence of depression and anxiety. The number of healthy, depression (**A**), and anxiety (**B**) diagnosed patients across the follow-up period. Depression and anxiety severity were diagnosed using LEVEL 2—Depression—Adult (PROMIS Emotional Distress—Depression—Short Form) and LEVEL 2—Anxiety—Adult (PROMIS Emotional Distress—Anxiety—Short Form), following scoring criteria; *n* = 35.

Based on DSM-5 scoring for depression and anxiety, we classified the severity of these symptoms (Figure 1). The graphical analysis illustrates changes in the proportion of patients diagnosed with depression and anxiety throughout the follow-up period. A substantial reduction in diagnosed depression and anxiety cases was observed after bariatric surgery, with some improvements post-abdominoplasty. This suggests that weight loss and body contouring procedures contribute to a reduction in anxiety severity. Overall, our findings highlight the positive psychological impact of bariatric surgery and abdominoplasty, demonstrating significant improvements in both depression and anxiety scores.

### 3.3. Self-Esteem and Body Image Perception

Self-esteem, measured by the Rosenberg Self-Esteem Scale, significantly improved following bariatric surgery. Mean scores increased from 19.19 ± 5.53 preoperatively to 21.31 ± 5.22 post-bariatric surgery (*p* < 0.001). However, no additional improvement was observed post-abdominoplasty. The score slightly declined to 20.09 ± 5.3, which was not significantly different from preoperative values (*p* = 0.56). This suggests that the major improvement in self-esteem was primarily due to weight loss rather than body contouring.

Body perception, assessed using the Body Shape Questionnaire (BSQ), improved significantly after both surgeries. Scores decreased from 124.7 ± 44.76 preoperatively to 82.83 ± 29.7 post-bariatric surgery (*p* < 0.001) and remained stable at 81.21 ± 27.65 post-abdominoplasty (*p* < 0.001 compared to preoperative). The difference between post-bariatric and post-abdominoplasty stages was not significant (*p* = 0.806).

Subjective aesthetic satisfaction, assessed with the Global Aesthetic Improvement Scale (GAIS), showed similar scores when comparing post-bariatric to preoperative appearance (median = 2 [IQR: 1–2]) and post-abdominoplasty to preoperative appearance (median = 2 [IQR: 1–2.5]). A score of 2 indicates “much improved.” The lack of statistical difference between these assessments (*p* = 0.635) suggests that patients did not perceive a significant additional aesthetic benefit from abdominoplasty (Table 4).

Overall, our data suggest that despite a significant change in body shape after abdominoplasty, most patients tend to value the effect of substantial weight loss rather than body contouring itself. Especially in the GAIS scale, patients were asked to assess the change in their body aesthetics compared to how they looked before bariatric surgery. It is also possible that the aesthetic outcome of both procedures, or especially abdominoplasty, did not meet patients’ expectations they had before starting surgical treatment of obesity.

### 3.4. Social Relationships

The Social Provisions Scale showed a modest but not statistically significant improvement in total social support following bariatric surgery, increasing from 75.97 ± 12.2 to 78.54 ± 9.32 (*p* = 0.058). Scores remained stable post-abdominoplasty at 78.25 ± 10.34 (*p* = 0.21 vs. preoperative; *p* = 0.51 vs. post-bariatric).

Subdomain analysis revealed that attachment significantly improved from 12 [10,11,12,13,14] preoperatively to 13 [12,13,14,15] post-bariatric surgery (*p* < 0.001) and to 14 [12,13,14,15] post-abdominoplasty (*p* < 0.05). Reliable alliance also improved significantly post-bariatric surgery (from 13 [12,13,14,15] to 14 [12,13,14,15], *p* < 0.05), but this change was not significant post-abdominoplasty (*p* = 0.079). No significant changes were observed in the other subdomains (Table 5).

### 3.5. Sexual Functioning

Sexual functioning, measured with the Changes in Sexual Functioning Questionnaire (CSFQ), improved significantly after bariatric surgery and was maintained after abdominoplasty. Total scores rose from 48 (IQR: 32.75–55) preoperatively to 52 (IQR: 39–59) post-bariatric surgery (*p* < 0.001) and remained stable afterward. All subdomains except sexual interest improved significantly post-surgery. The lack of change in sexual interest may be explained by preoperative scores already being comparable to healthy controls [21]. Interestingly, desire frequency increased from 6 [4,5,6,7,8] to 7 [5,6,7,8] post-bariatric surgery (*p* < 0.05), but declined back to 6 [4,5,6,7,8] post-abdominoplasty (*p* = 0.514), with a near-significant difference compared to the post-bariatric stage (*p* = 0.051). These results indicate that, despite improvements in body image and psychological well-being, abdominoplasty did not lead to further gains in sexual functioning, which aligns with the observed trends in self-esteem and aesthetic perception (Table 6).

## 4. Discussion

With obesity rates continuing to rise, the burden of this global health issue has never been greater. However, advances in treatment, including bariatric surgery and the emergence of new, effective anti-obesity medications, such as GLP-1 agonists, are enabling more individuals to achieve significant weight loss [3,24,25]. As a result, the number of patients seeking solutions for excess skin following major weight reduction is steadily rising.

Aesthetic surgery is becoming increasingly accessible and popular among these patients. Procedures such as abdominoplasty and lower body lifts not only remove excessive loose skin but also enhance both appearance and overall quality of life [26,27]. Beyond cosmetic benefits, these surgeries contribute to improved mobility, hygiene, and psychological well-being. Despite the growing number of body contouring procedures being performed, reliable scientific evidence on their impact, particularly on quality of life and sexual well-being, remains limited. Existing studies often rely on varied, non-standardized, or investigator-created questionnaires, making direct comparisons between findings challenging. That is why we aimed to use the most widely recognized and validated psychological assessment tools, such as DSM-5 criteria, PHQ-9, and the RSES. Additionally, we incorporated other measures specifically designed to evaluate the impact of bariatric and body contouring surgeries on quality of life and sexual health. To our knowledge, this is the first study to comprehensively assess how bariatric surgery, followed by abdominoplasty, affects quality of life, psychological well-being, and sexual health. We analyzed data from patients at three key stages: before undergoing sleeve gastrectomy, after sleeve gastrectomy, and after subsequent abdominoplasty. By comparing these datasets, we aimed to gain a deeper understanding of the long-term benefits and challenges faced by patients who undergo this transformative surgical process. We also wanted to determine whether aesthetic surgery provides a measurable additional benefit.

Bariatric surgery led to significant improvements in overall psychological health, as reflected in the reduction in DSM-5 scores and PHQ-9 depression scores. Interestingly, abdominoplasty was associated with additional mental health benefits, as indicated by further decreases in DSM-5 total scores and specific domains such as anxiety, sleep problems, somatic symptoms, and substance use. These findings suggest that while bariatric surgery is the primary driver of psychological improvement, body contouring surgery contributes to further emotional stabilization. The observed improvement in the general quality of life after abdominoplasty seems to be in line with most literature [26,27,28,29,30,31,32,33,34,35]. A systematic review and meta-analysis by Toma et al. demonstrated that body contouring surgery significantly improves quality of life in post-bariatric patients, particularly in physical, social, and psychological functioning [36].

A novel finding in our study is the significant reduction in anxiety symptoms following abdominoplasty, as measured by the LEVEL 2—Anxiety questionnaire for adults developed by the PROMIS Health Organization. In contrast, previous studies by de Zwaan et al. and Saariniemi et al. did not observe a significant effect of abdominoplasty on anxiety symptoms [27,34]. However, methodological differences limit comparison. For example, de Zwaan et al. compared two groups of post-bariatric patients, one who underwent abdominoplasty and one who did not, using the 7-item Generalized Anxiety Disorder Scale [27]. Saariniemi et al. assessed non-post-bariatric patients undergoing abdominoplasty for aesthetic reasons using Raitasalo’s modification of the Beck Depression Inventory (RBDI), which includes only a single item related to anxiety [34]. As a result, both studies had limited ability to assess anxiety severity. Nonetheless, they observed a slight, non-significant reduction in anxiety symptoms, which aligns with our results. In our study, abdominoplasty showed a near-significant improvement in depressive symptoms and diagnosed depression cases, likely limited by sample size. Only two studies to date have reported a clear antidepressant effect of abdominoplasty. Saariniemi et al. observed improved RBDI scores and reduced depression severity and diagnoses [34]. Papadopulos et al. similarly found a decrease in depressive symptoms in non-bariatric patients assessed with the PHQ-4 [29]. In contrast, de Zwaan et al. found no significant difference in PHQ-9 scores between post-bariatric patients who underwent abdominoplasty and those who did not [27].

Contrary to our expectations, abdominoplasty did not lead to additional improvements in body perception beyond those achieved by bariatric surgery. BSQ scores improved significantly following bariatric surgery but remained unchanged after abdominoplasty, suggesting that weight loss alone is the primary factor in body image change. Similarly, there was no significant difference in GAIS scores before and after abdominoplasty. Although patients reported improved aesthetics compared to the pre-bariatric state, abdominoplasty did not add further perceived improvement. Likewise, self-esteem, as measured by the RSES, improved significantly post-bariatric surgery but did not improve further after abdominoplasty. This may be due to ongoing dissatisfaction with untreated body areas or the overwhelming impact of major weight loss compared to contouring procedures. In contrast, Papadopulos et al. found higher self-esteem scores post-abdominoplasty compared to the general population [30]. However, their study did not assess preoperative values, so the source of this improvement remains unclear. Uimonen et al. reported that patients undergoing abdominoplasty after major weight loss had lower health-related quality of life than gender-, age-, and BMI-adjusted general population [37]. Bolton et al. found improved appearance satisfaction after abdominoplasty but no change in self-esteem, which supports our findings [38]. This suggests that while body contouring may enhance satisfaction with specific aesthetic outcomes, it does not necessarily translate into broader improvements in self-worth. Similarly, Berkane et al. found no effect of abdominoplasty on self-esteem in patients following major weight loss, further supporting the notion that body contouring does not necessarily enhance overall self-perception [26]. Conversely, de Zwaan et al. reported higher self-esteem in post-bariatric patients who underwent abdominoplasty compared to those who did not [27]. However, they also noted considerable variability in satisfaction, with some individuals remaining dissatisfied with their body aesthetics despite surgery. This variability may reflect differences in patient expectations or preexisting psychological factors that influence post-surgical self-perception. Taken together, these studies suggest that while abdominoplasty can refine body aesthetics, its psychological impact varies among individuals and may not consistently improve self-esteem, especially when residual concerns about body image persist. It is worth noting that dissatisfaction with body image and lack of self-confidence are among the most common reasons to undergo body contouring surgery [35,39]. This underscores the importance of managing patient expectations regarding abdominoplasty, ensuring they understand that while it can address localized concerns, it may not lead to a comprehensive improvement in body image or self-esteem.

Regarding social support, our study showed modest improvement in attachment and perceived reliable support after bariatric surgery, but no significant change after abdominoplasty. This indicates that major lifestyle and weight-related transformations are more influential in reshaping social relationships than aesthetic procedures alone.

Sexual functioning also improved following bariatric surgery, particularly in domains such as pleasure, arousal, and orgasm, as assessed with the CSFQ. However, no further improvement was observed after abdominoplasty. Although some studies suggest that body contouring can enhance sexual confidence, our findings point to weight loss as the main driver of improved sexual well-being [38]. Berkane et al. found a temporary improvement in sexual functioning post-abdominoplasty using the Female Sexual Function Index, which diminished after 12 months [26]. This transient benefit aligns with our findings and those of Toma et al., whose meta-analysis found no effect of abdominoplasty on body image, sexual function, pain, or self-esteem in post-bariatric patients [36].

## 5. Limitations

Several limitations should be considered when interpreting our findings. First, the retrospective design and reliance on patient recall may introduce recall bias in self-reported outcomes. Additionally, the sample size was relatively small, which may have limited the statistical power to detect subtle effects of abdominoplasty. The limited sample size also precluded multivariate analysis to account for potential confounding variables such as time since surgery, age, gender, marital status, or comorbid psychiatric conditions, which may influence psychological outcomes.

Furthermore, this was a single-center study, which limits the generalizability of the results. Although 135 patients were invited, the majority did not return the questionnaires, despite initially expressing interest. The low response rate (25.9%) raises concerns regarding selection bias. It is possible that individuals who returned the questionnaires were more engaged, had more favorable experiences, or differed psychologically from non-respondents. This could lead to an overestimation of the psychological benefits observed and limit the applicability of our results.

It is also worth noting that the survey included a total of 26 individual questionnaires, which may have discouraged patients from completing and returning them, despite initial interest. Lastly, the study population was predominantly female, which means the results may not be equally representative of male patients.

## 6. Conclusions

This study highlights the psychological benefits of abdominoplasty following bariatric surgery, particularly in reducing anxiety and improving overall psychological well-being. However, its impact on body perception, self-esteem, social relationships, and sexual functioning appears limited. While abdominoplasty remains an important component of post-bariatric care, its effects should be regarded as complementary rather than transformative. Our findings emphasize the importance of setting realistic expectations for patients considering body contouring surgery. It is crucial to ensure that individuals understand that while abdominoplasty can address localized concerns such as excess abdominal skin, it may not lead to a complete transformation in body image or self-worth.

## 7. Future Directions

Future research should employ prospective longitudinal designs and include larger, more diverse cohorts to better evaluate the long-term effects of body contouring procedures. Additionally, studies should investigate the impact of combined or sequential aesthetic surgeries, such as breast lifts, thigh lifts, or arm contouring, to provide a more comprehensive understanding of how these interventions influence body perception, psychological health, and social functioning in patients following major weight loss.

## Figures and Tables

**Table 1:** The baseline characteristics of patients. Total weight lost (%TWL) refers to % of initial body weight lost after bariatric surgery. NA—not acquired. Results are presented as mean ± SD.

	Respondents (*n* = 35)	Non-Respondents (*n* = 100)
Response rate	35/135 (25.9)	
Characteristics	Value (%)	Value (%)
Age (years)	51 ± 8.9	46 ± 11.1
Gender		
Female	26 (74.3)	83 (83)
Male	9 (25.7)	17 (17)
Weight (kg)	131.5 ± 26	147.5 ± 27.1
BMI	48.9 ± 7.8	48.9 ± 8.1
%TWL	30 ± 9.6	NA
Waist circumference	91.9 ± 19.5	NA
Hips circumference	115 ± 14.9	NA

**Table 2:** The effect of bariatric surgery and subsequent abdominoplasty on psychological well-being assessed in PHQ-9 and DSM-5 questionnaires. DSM-5-TR Level 1 Cross-Cutting Symptom Measures were presented as total scores (DSM-5) and summarized scores for each mental health domain assessed in the test according to the scoring instructions. Results are presented as median and IQR. Pairwise comparisons were analyzed with a paired Wilcoxon test; *n* = 35.

	Preoperative	Post-Bariatric	P (Preoperative vs. Post-Bariatric)	Post-Abdominoplasty	P (Preoperative vs. Post-Abdominoplasty)	P (Post-Bariatric vs. Post-Abdominoplasty)
PHQ-9	9 (4.25–14)	4 (2–7)	*<0.001*	3 (1–6)	*<0.001*	*0.052*
DSM-5	16 (11–25.75)	12 (5–14)	*<0.001*	8 (3–12)	*<0.001*	*<0.01*
Depression	3 (2–4)	2 (0–2)	*<0.001*	2 (0–2)	*<0.001*	*0.709*
Anger	1 (1–2)	1 (0–1)	*<0.001*	0 (0–1)	*<0.001*	*0.283*
Mania	2 (1–3)	2 (1–4)	*0.294*	1 (0–3)	*0.347*	*<0.01*
Anxiety	3 (1.25–5.75)	2 (0–3)	*<0.001*	1 (0–2)	*<0.001*	*0.191*
Somatic symptoms	2 (0–5)	1 (0–2)	*<0.01*	0 (0–1)	*<0.001*	*<0.05*
Suicidal ideation	0 (0–0)	0 (0–0)	*>0.999*	0 (0–0)	*0.5*	*0.5*
Psychosis	0 (0–0)	0 (0–0)	*0.5*	0 (0–0)	*> 0.999*	*>0.999*
Sleep problems	1 (0–2)	1 (0–1)	*<0.05*	0 (0–1)	*<0.001*	*<0.05*
Memory	0 (0–1)	0 (0–0)	*<0.01*	0 (0–0)	*<0.01*	*>0.999*
Repetitive thoughts and behaviors	0 (0–0.75)	0 (0–0)	*<0.05*	0 (0–0)	*<0.05*	*>0.999*
Dissociation	0 (0–0)	0 (0–0)	*<0.05*	0 (0–0)	*0.094*	*0.5*
Personality functioning	0 (0–2)	0 (0–1)	*<0.01*	0 (0–0)	*<0.01*	*>0.999*
Substance use	1 (0–2.75)	1 (0–2)	*0.072*	0 (0–2)	*<0.01*	*0.469*

**Table 3:** The effect of bariatric surgery and subsequent abdominoplasty on depression and anxiety. Level 2—Depression—Adult (PROMIS Emotional Distress—Depression—Short Form) and Level 2—Anxiety—Adult (PROMIS Emotional Distress—Anxiety—Short Form) were used to assess depression and anxiety severity. Results are presented as median and IQR. Pairwise comparisons were analyzed with a paired Wilcoxon test; *n* = 35.

	Preoperative	Post-Bariatric	P (Preoperative vs. Post-Bariatric)	Post-Abdominoplasty	P (Preoperative vs. Post-Abdominoplasty)	P (Post-Bariatric vs. Post-Abdominoplasty)
DSM-5-TR Level 2—Depression	10.5 (5–17.5)	4 (2–7)	*<0.001*	3 (1–6)	*<0.001*	*0.052*
DSM-5—PROMIS Level 2—Anxiety	4 (0–12.25)	1 (0–5)	*<0.01*	1 (0–3)	*<0.001*	*<0.05*

**Table 4:** The effect of bariatric surgery and subsequent abdominoplasty on self-esteem and body image perception. Self-esteem was assessed using the Rosenberg Self-Esteem Scale. Results are presented as mean ± SD. Pairwise comparisons were analyzed with a paired *t*-test. Own body perception was assessed using the Body Shape Questionnaire. Results are presented as mean ± SD. Pairwise comparisons were analyzed with a paired *t*-test. Subjective aesthetic improvement after bariatric surgery and abdominoplasty was assessed by each patient individually using the Global Aesthetic Improvement Scale (GAIS). Results are presented as median and IQR. Pairwise comparisons were analyzed with a paired Wilcoxon test; *n* = 35.

	Preoperative	Post-Bariatric	P (Preoperative vs. Post-Bariatric)	Post-Abdominoplasty	P (Preoperative vs. Post-Abdominoplasty)	P (Post-Bariatric vs. Post-Abdominoplasty)
Rosenberg Self-Esteem Scale	19.19 ± 5.53	21.31 ± 5.22	*<0.001*	20.09 ± 5.3	*0.56*	*0.094*
Body Shape Questionnaire	124.7 ± 44.76	82.83 ± 29.7	*<0.001*	81.21 ± 27.65	*<0.001*	*0.806*
GAIS		2 (1–2)		2 (1–2.5)		*0.635*

**Table 5:** The effect of bariatric surgery and subsequent abdominoplasty on social relationships. Social relationships were assessed using the Social Provisions Scale. Total scores are presented as mean ± SD. Pairwise comparisons were analyzed with a paired *t*-test. Particular subdomain scores are presented as median and IQR. Pairwise comparisons were analyzed with a paired Wilcoxon test; *n* = 35.

	Preoperative	Post-Bariatric	*P (Preoperative* vs. *Post-Bariatric)*	Post-Abdominoplasty	*P (Preoperative* vs. *Post-Abdominoplasty)*	*P (Post-Bariatric* vs. *Post-Abdominoplasty)*
Social Provisions Scale	75.97 ± 12.2	78.54 ± 9.32	*0.058*	78.25 ± 10.34	*0.21*	*0.51*
Guidance	13 (11–16)	13 (12–15)	*0.354*	13 (12–16)	*0.612*	*0.612*
Reassurance	12 (11–13)	12 (11–13)	*0.681*	12 (11–13)	*0.381*	*0.623*
Social	12 (11–14)	13 (12–14)	*0.091*	13 (12–14.75)	*0.418*	*0.489*
Attachment	12 (10–14)	13 (12–15)	*<0.001*	14 (12–15)	*<0.05*	*0.756*
Nurturance	13 (12–15)	14 (12–14)	*0.426*	13.5 (12–14.75)	*0.448*	*0.581*
Reliable	13 (12–15)	14 (12–15)	*<0.05*	14 (12–15)	*0.079*	*0.354*

**Table 6:** The effect of bariatric surgery and subsequent abdominoplasty on sexual functioning. Sexual activity was assessed using the Changes in Sexual Functioning Questionnaire (CSFQ). Both the total CSFQ score and each subdomain result were analyzed according to the scoring instructions. Normally distributed results are presented as mean ± SD, and pairwise comparisons were analyzed with a paired *t*-test. Non-normally distributed results are presented as median and IQR, and pairwise comparisons were analyzed with a paired Wilcoxon test; *n* = 35.

	Preoperative	Post-Bariatric	*P (Preoperative* vs. *Post-Bariatric)*	Post-Abdominoplasty	*P (Preoperative* vs. *Post-Abdominoplasty)*	*P (Post-Bariatric* vs. *Post-Abdominoplasty)*
CSFQ	48 (32.75–55)	52 (39–59)	*<0.001*	52 (41.5–58.25)	*<0.01*	*0.819*
Pleasure	3 (2–4)	4 (3–4)	*<0.001*	4 (3–4)	*<0.001*	*>0.999*
Desire/Frequency	6 (4–8)	7 (5–8)	*<0.05*	6 (4–8)	*0.514*	*0.051*
Desire/Interest	7.47 ± 2.95	8.12 ± 2.84	*0.258*	8.57 ± 2.62	*0.071*	*0.141*
Arousal	10 (6.25–12)	12 (9–13)	*<0.01*	12 (9–13.25)	*<0.01*	*0.617*
Orgasm	10.5 (6–12.75)	12 (8.5–14)	*<0.001*	12 (10–14)	*<0.01*	*0.853*

## Data Availability

The raw data supporting the conclusions of this article will be made available by the authors on request.
